# Role of mTOR-Regulated Autophagy in Synaptic Plasticity Related Proteins Downregulation and the Reference Memory Deficits Induced by Anesthesia/Surgery in Aged Mice

**DOI:** 10.3389/fnagi.2021.628541

**Published:** 2021-04-16

**Authors:** Sunan Gao, Siyu Zhang, Hongmei Zhou, Xiaoyan Tao, Yunjian Ni, Daqing Pei, Shuai Kang, Weiwei Yan, Jian Lu

**Affiliations:** ^1^Department of Anesthesiology, Zhejiang Chinese Medical University, Hangzhou City, China; ^2^Department of Anesthesiology, The Second Hospital of Jiaxing, The Second Affiliated Hospital of Jiaxing University, Jiaxing City, China; ^3^Department of Nursing, The Second Hospital of Jiaxing, The Second Affiliated Hospital of Jiaxing University, Jiaxing City, China

**Keywords:** rapamycin, mTOR, postoperative cognitive dysfunction, aged, synaptic plasticity

## Abstract

Postoperative cognitive dysfunction increases mortality and morbidity in perioperative patients and has become a major concern for patients and caregivers. Previous studies demonstrated that synaptic plasticity is closely related to cognitive function, anesthesia and surgery inhibit synaptic function. In central nervous system, autophagy is vital to synaptic plasticity, homeostasis of synapticproteins, synapse elimination, spine pruning, proper axon guidance, and when dysregulated, is associated with behavioral and memory functions disorders. The mammalian target of rapamycin (mTOR) negatively regulates the process of autophagy. This study aimed to explore whether rapamycin can ameliorate anesthesia/surgery-induced cognitive deficits by inhibiting mTOR, activating autophagy and rising synaptic plasticity-related proteins in the hippocampus. Aged C57BL/6J mice were used to establish POCD models with exploratory laparotomy under isoflurane anesthesia. The Morris Water Maze (MWM) was used to measure reference memory after anesthesia and surgery. The levels of mTOR phosphorylation (p-mTOR), Beclin-1 and LC3-II were examined on postoperative days 1, 3 and 7 by western blotting. The levels of synaptophysin (SYN) and postsynaptic density protein 95 (PSD-95) in the hippocampus were also examined by western blotting. Here we showed that anesthesia/surgery impaired reference memory and induced the activation of mTOR, decreased the expression of autophagy-related proteins such as Beclin-1 and LC3-II. A corresponding decline in the expression of neuronal/synaptic, plasticity-related proteins such as SYN and PSD-95 was also observed. Pretreating mice with rapamycin inhibited the activation of mTOR and restored autophagy function, also increased the expression of SYN and PSD-95. Furthermore, anesthesia/surgery-induced learning and memory deficits were also reversed by rapamycin pretreatment. In conclusion, anesthesia/surgery induced mTOR hyperactivation and autophagy impairments, and then reduced the levels of SYN and PSD-95 in the hippocampus. An mTOR inhibitor, rapamycin, ameliorated anesthesia/surgery-related cognitive impairments by inhibiting the mTOR activity, inducing activation of autophagy, enhancing SYN and PSD-95 expression.

## Introduction

Postoperative cognitive dysfunction (POCD) is a complication of the central nervous system after anesthesia and surgery, mainly manifested in the deterioration of cognitive function, especially learning and memory, attention (Abildstrom et al., 2000; Berger et al., 2015; Steinmetz and Rasmussen, 2016; Holmgaard et al., 2019). It may last for days, months or even years, result in the increase of hospitalization expense and extension of hospitalization time, and markedly impair postoperative recovery and increase morbidity and mortality (Steinmetz et al., 2009; Bilotta et al., 2010; Quan et al., 2019). Although POCD is an important clinical issue, the pathogenesis of POCD remains to be fully elucidated. Furthermore, clarifying its pathogenesis can help to prevent its occurrence.

Mammalian target of rapamycin (mTOR) is a central regulator of protein synthesis in neurons. Several studies have revealed that mTOR signaling may be correlated to neurodegenerative diseases (Garelick and Kennedy, 2011; Heras-Sandoval et al., 2014), including Parkinson disease, Huntington’s disease, Alzheimer’s disease (Bockaert and Marin, 2015). In Alzheimer’s disease, the inhibition of mTOR by rapamycin ameliorates the synthesis of β-amyloid and tau protein and improves learning and memory abilities (Spilman et al., 2010). mTOR is necessary for phagophore and autophagosome formation. In the central nervous system, autophagy and its regulation by mTOR (Costa-Mattioli and Monteggia, 2013) are critical for maintaining neuronal functions (Kulkarni and Maday, 2018), such as synaptic remodeling and plasticity associated with long-term memory formation (Shehata and Inokuchi, 2014). Montesinos et al. (2018) have demonstrated that autophagy is impaired by increasing autophagy inhibitor mTOR. Inhibition of mTOR, by rapamycin, reestablishes the LC3-II levels and restores the levels of synaptic plasticity-related proteins including PSD-95, SHANK3 and p62. These suggest that mTOR-regulated autophagy system plays an important role in the synaptic plasticity-related proteins alterations. SYN and PSD-95 are synaptic functional proteins, which are related to synaptic plasticity (Pan et al., 2017; Liu et al., 2018; Ma et al., 2019). Therefore, in the present study, we explored that whether the modulation of the autophagy activation, by inhibiting mTOR with rapamycin administration, was capable of restoring alterations in the decline of synaptic plasticity-related proteins and the cognitive impairment induced by anesthesia/surgery.

## Materials and Methods

Ethical approval for this study was provided by the Animal Care and Use Committee of the Second Affiliated Hospital of Jiaxing University. All animal procedures were performed in accordance with the National Institutes of Health animal care guidelines.

### Animals, Anesthesia/Surgery and Procedure Pharmacological Treatment

Anesthesia was prepared using C57BL/6Jmice (18-month old, male) were provided by Hunan SJA Laboratory Animal Company, Limited. The animals were housed under controlled conditions (temperature of 22 ± 2°C, 12 h light/12 h dark cycle) with access to food and water *ad libitum*. All mice were allowed to adapt to their new environment for 7 days before beginning the experiments.

The mice were randomly divided into four groups: control group (mice received injections of vehicle without anesthesia/surgery), control+rapa group (mice received rapamycin pretreatment without anesthesia/surgery), anesthesia/surgery group (mice received anesthesia/surgery with injections of vehicle) and anesthesia/surgery+rapa group (mice received anesthesia/surgery with rapamycin pretreatment). Exploratory laparotomy was performed under isoflurane anesthesia (induced with 4.0% isoflurane and maintained with 2.0% isoflurane in 0.30 FiO_2_). First, a midline laparotomy (3 cm in length) was performed. Second, the sterile cotton swabs were dipped in the wet normal saline, and the abdominal organs were explored successively. The exploration in the abdominal cavity lasted for 3 min and the exposure lasted for 3 min. Aseptic techniques were used during the entire procedure, which required approximately 30 min. Third, around the incision, 0.1% lidocaine infiltration was used as postoperative analgesia, and then the wound was closed by 5–0 Vicryl sutures. The wound was covered with bacitracin ointment. The mice were gently restrained to a heating pad (37°C) using paper tape, and the whole procedure lasted 20 min. The dose of rapamycin used was selected based on previous studies (Tang et al., 2014). The mice received intraperitoneal injections of 0.5 mg/kg/day rapamycin for 3 days prior to undergoing exploratory laparotomy.

We used the Morris Water Maze (MWM) to test the reference memory of aged mice. Mice were euthanized on days 1, 3 and 7 days after anesthesia/surgery in each group. Hippocampus tissue was dissected for western blot analysis, and stored at −80°C.

### Morris Water Maze (MWM)

The MWM test was conducted in a large circular pool (110 cm in diameter and 30 cm in depth) made of white plastic. The pool was filled with water to a depth of 35 cm (maintained at 23–25°C), covering a transparent circular platform 10 cm in diameter. The platform was submerged approximately 1.0 cm below the surface of the water. The pool was situated in a room with diffuse lighting and was surrounded by curtains. The curtains were white and had distinct cues painted on them. The position of the cues remained unchanged throughout the task. The swimming activity of each mouse was monitored and tracked *via* a television camera (HIK VISION Company, Limited, Hangzhou, China) mounted overhead, which relayed information, including variables such as latency to the platform, total distance traveled and time spent in each quadrant, to a video-tracking system (RWD Company, Limited, Shenzhen, China).

After the anesthesia/surgical procedure, training was conducted for 4 days for the reference memory test. Each day, a trial was initiated by placing each mouse in the water facing the pool wall in one of the four quadrants, which was randomly selected. For each training trial, the mouse was allowed to swim for 60 s to find the hidden platform. When successful, the mouse was allowed a 30-s rest period on the platform. If the mouse failed to find the platform within 60 s, it was gently guided to the platform and allowed a 30-s rest period on the platform. On the fourth day of training, the latency of each group was analyzed. The probe trials were conducted after the training trials. In this test, the platform was removed from the pool and the mice were allowed to search for it for 60 s. The time spent in the target quadrant, where the platform is placed, was analyzed.

### Western Blotting

The hippocampal tissues were homogenized in RIPA buffer containing protease and phosphatase inhibitors, and then centrifuged at 4°C at 12,000 *g* for 20 min. The concentration of protein in the supernatants was determined using a Bradford protein assay kit (Servicebiotechnology Company, Limited, Wuhan, China). Equal quantities of the protein samples were denatured at 100°C for 15 min, and were then separated by10% sodium dodecyl sulfate-polyacrylamide gel electrophoresis and transferred electrophoretically onto a polyvinylidene fluoride membrane (Millipore). The membranes were blocked using 5%-TBST for 60 min and then incubated with the following primary antibodies: mTOR phosphoS2448 (p-mTOR, 1:1,000; Abcam), postsynaptic density protein 95 (PSD-95, 1:1,000; Abcam) and synaptophysin (SYN, 1:1,000; Abcam) at 4°C overnight. Following the membranes were washed three times (5 min each) in TBST and incubated with secondary antibody for 60 min at room temperature. The membranes were detected using an enhanced chemiluminescence system (CLINX, China) and analyzed using AlphaEaseFC (Alpha Innotech).

### Quantitative Real-Time Polymerase Chain Reaction (qRT-PCR)

Quantitative real-time PCR was performed on the ABI7900 (Illumina) with FastStart Universal SYBR Green Master(Rox) (Roche). cDNA was synthesized from RNA (65°C for 5 min, 42°C for 60 min, and 70°C for 5 min) using RevertAid First Strand cDNA Synthesis Kit (Thermo). The DNA was amplified (95°C for 10 min; and 40 cycles of 95°C for 15 s, 60°C for 60 s). The sequences of specific primers are summarized in Table 1. The expression level of each gene was calculated based on the comparative threshold cycle value (Ct) method, using the formula 2 − ΔΔCt (ΔΔCt = ΔCt sample − ΔCt reference). The final results were expressed as normalized fold values relative to the control group.

**Table 1:** The primer sequence.

Name	Forward	Reverse	Size (bp)
GAPDH	5′-CCTCGTCCCGTAGACAAAATG-3′	5′-TGAGGTCAATGAAGGGGTCGT-3′	133
SYN	5′-GGCGGCACTTCTGTCATCAA-3′	5′-AATGACACCTCCCAGCACTTC-3′	225
PSD-95	5′-CGATTACCACTTTGTCTCCTCCC-3′	5′-ACGGATGAAGATGGCGATAGG-3′	220

GAPDH, glyceraldehyde 3-phosphate dehydrogenase; SYN, synaptophysin; PSD-95, postsynaptic density protein 95.

### Statistical Analysis

Data are presented as the mean ± SEM. Statistical analyses were performed with GraphPad Prism 6.0 (GraphPad Software Inc., USA). A two-way ANOVA was used to analyze the water maze escape latency and average speed, and relative protein levels of p-mTOR, Beclin-1 and LC3-II, SYN and PSD-95. A one-way ANOVA or unpaired *t*-test was used to analyze the probe quadrant trial data, probe test data. The data were checked for normality before running ANOVA. *p* < 0.05 was considered to indicate a statistically significant difference.

## Results

### Anesthesia and Surgery Impaired Reference Memory in Aged Mice

**Figure 1:**
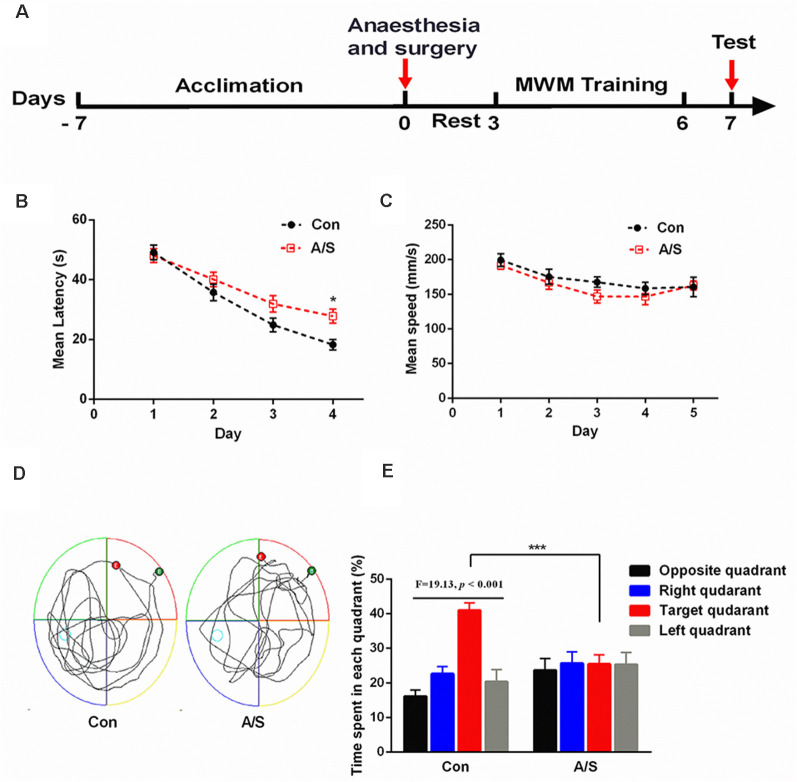
Anesthesia and surgery impaired reference memory in aged mice. **(A)** Schematic timeline of the experimental paradigm of surgery procedure and Morris water maze test. After the anesthesia/surgical procedure, training was conducted for 4 days followed by probe tests on day 7. **(B)** Escape latency to reach the hidden platform during the 4-day training; the mean latency of the anesthesia/surgery group was significantly prolonged on day 4 than the control. **(C)** Average swimming speed; there was no significant difference between the two groups. **(D)** Representative exploratory path of two groups of mice in the probe test. **(E)** The percentage of time spent in the target quadrant during the probe test; there was significant difference between the control group and the anesthesia/surgery group. The percentage of time spent in the target quadrant was significantly higher than the other quadrants in control mice. Data are mean ± SEM (*n* = 12 per group). ***p < 0.001 vs. Con. Con, control group; A/S, anesthesia/surgery group.

To determine whether anesthesia/surgery produced cognitive dysfunction in aged mice, we used the MWM to evaluate learning and memory after exploratory laparotomy. As shown in Figure 1A, mice were acclimated to the environment for 7 days before the experiments were conducted. Exploratory laparotomy was performed under isoflurane anesthesia, the mice were allowed to rest for 2 days after the surgery. The training course for the water maze lasted for 4 days, probe tests were conducted on day 7 (Figure 1A). Two-way ANOVA revealed that the mean latency of the mice from the anesthesia/surgery group was significantly prolonged on the fourth day of training than the control mice (*F*_(1,88)_ = 8.686; *p* < 0.01; Figure 1B). Two-way ANOVA revealed that the average swimming speed during the training and probe tests were not significantly different between the two groups (Figure 1C), this excluded the effects of motor and perceptual abilities on spatial learning and memory after anesthesia/surgery. In the probe test, unpaired *t*-test revealed that swimming time of the anesthesia/surgery group in the target quadrant was significantly shorter than the control group (*t* = 4.422; *p* < 0.001; exploratory path of two groups of mice in the probe test; Figure 1D); ANOVA revealed that the time spent in the target quadrant was significantly higher than the other quadrants in control mice (*F* = 19.13; *p* < 0.001; Figure 1E). Anesthesia/surgery did not impair reference memory function in young mice (Zhao et al., 2016). These results suggested that anesthesia/surgery impaired reference memory in aged mice.

### Anesthesia and Surgery Induced the Upregulation of p-mTOR and Decreased LC3-II and Beclin-1 Expression in the Hippocampus

**Figure 2:**
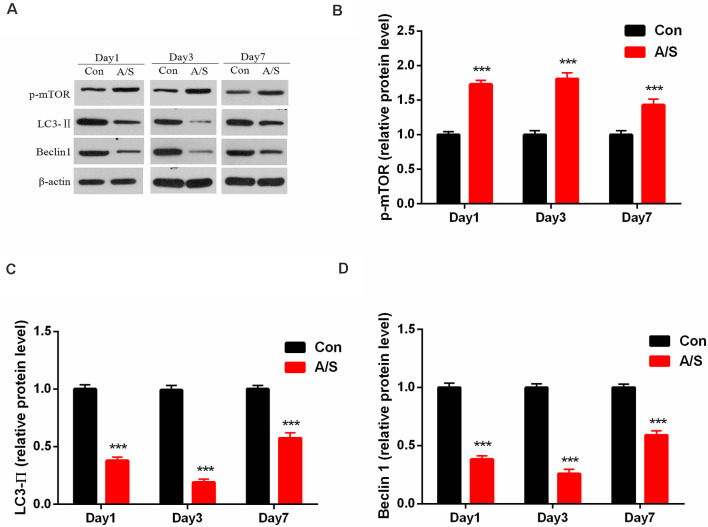
Anesthesia and surgery increased the activation of mammalian target of rapamycin (mTOR) and decreased LC3-II and Beclin-1 levels in the hippocampus. **(A)** Representative western blot illustrating p-mTOR, LC3-II and Beclin-1 levels in the hippocampus on days 1, 3 and 7 after surgery. **(B)** Anesthesia/surgery increased p-mTOR expression in the hippocampus on days 1, 3 and 7 after anesthesia/surgery. **(C,D)** Anesthesia/surgery reduced LC3-II and Beclin-1 levels in the hippocampus on days 1, 3 and 7 after anesthesia/surgery. Data are mean ± SEM (*n* = 6 per group). ***p < 0.001 vs. Con. Con, control group; A/S, anesthesia/surgery group.

To investigate the effect of anesthesia/surgery on p-mTOR, LC3-II and Beclin-1 levels in the hippocampus, the present study detected the levels of p-mTOR, LC3-II and Beclin-1 levels in the homogenates of the hippocampal tissue using western blot analysis (Figure 2A). Two-way ANOVA revealed that anesthesia/surgery increased the production of p-mTOR (*F*_(1,30)_ = 145.9; *p* < 0.001) and decreased LC3-II (*F*_(1,30)_ = 414.1; *p* < 0.001) and Beclin-1 expression (*F*_(1,30)_ = 448.8; *p* < 0.001) on days 1, 3 and 7 after operation, compared with the control group, as shown in Figures 2B–D. These data indicated that anesthesia/surgery increased the expression of p-mTOR and decreased the levels of LC3-II and Beclin-1 on days 1, 3 and 7 after anesthesia/surgery.

### Anesthesia and Surgery Reduced SYN and PSD-95 Levels in the Hippocampus

**Figure 3:**
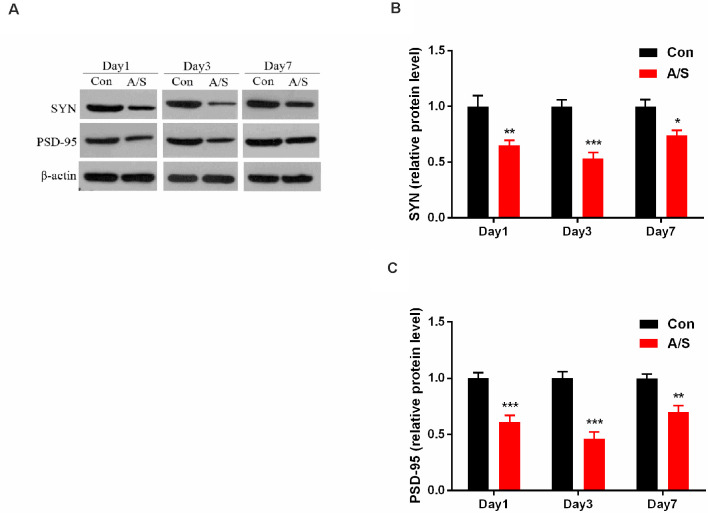
Anesthesia and surgery decreased synaptophysin (SYN) and postsynaptic density protein 95 (PSD-95) levels in the hippocampus. **(A)** Representative western blot illustrating SYN and PSD-95 levels in the hippocampus on days 1, 3 and 7 after anesthesia/surgery. **(B,C)** Anesthesia/surgery reduced SYN and PSD-95 expression in the hippocampus on days 1, 3 and 7 after anesthesia/surgery. Data are mean ± SEM (*n* = 6 per group). ***p < 0.001, **p < 0.01, *p < 0.05 vs. Con. Con, control group; A/S, anesthesia/surgery group.

In order to investigate the effect of anesthesia/surgery on the production of SYN and PSD-95, the present study detected the levels of SYN and PSD-95 in the homogenates of the hippocampal tissue using western blot analysis (Figure 3A). Two-way ANOVA revealed that anesthesia/surgery decreased the production of SYN (*F*_(1,30)_ = 47.78; *p* < 0.001) and PSD-95 (*F*_(1,30)_ = 85.89; *p* < 0.001) on days 1, 3 and 7 after operation, compared with the control group, as shown in Figures 3B,C. These data indicated that anesthesia/surgery decreased the levels of SYN and PSD-95 on days 1, 3 and 7 after anesthesia/surgery.

### Rapamycin Treatment Inhibited the Abnormal p-mTOR and Increased LC3-II and Beclin-1 Levels in the Hippocampus

**Figure 4:**
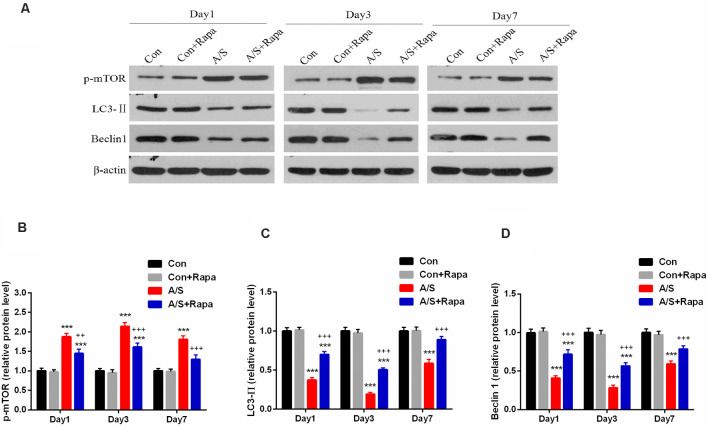
Rapamycin treatment inhibited the abnormal p-mTOR and increased LC3-II and Beclin-1 levels in the hippocampus. **(A)** Representative western blot illustrating the effects of rapamycin on the anesthesia/surgery-induced changes in hippocampal p-mTOR, LC3-II and Beclin-1 levels on days 1, 3 and 7 after anesthesia/surgery. **(B)** Rapamycin reversed anesthesia/surgery-induced increase in p-mTOR expression in the hippocampus on days 1, 3 and 7 after anesthesia/surgery. **(C,D)** Rapamycin reversed anesthesia/surgery-induced decrease in LC3-II and Beclin-1 levels in the hippocampus on days 1, 3 and 7 after anesthesia/surgery. Data are mean ± SEM (*n* = 6 per group). ***p < 0.001 vs. Con. ^+++^*p* < 0.001, ^++^*p* < 0.01 A/S. Con, control group; Con+Rapa, control group with rapamycin pretreatment; A/S, anesthesia/surgery group; A/S+Rapa, anesthesia/surgery group combined with rapamycin pretreatment.

In order to investigate whether the hyperactivation of p-mTOR was involved in the reduction of LC3-II and Beclin-1 levels, the present study examined the effect of rapamycin on the levels of LC3-II and Beclin-1 in the homogenates of the hippocampal tissue using western blot analysis (Figure 4A). It was found that rapamycin significantly increased the production of LC3-II (*F*_(3,60)_ = 160.9; *p* < 0.001) and Beclin-1 (*F*_(3,60)_ = 100.4; *p* < 0.001), and attenuated the levels of p-mTOR (*F*_(3,60)_ = 85.97; *p* < 0.001) on days 1, 3 and 7 after operation by using two-way ANOVA, compared with the anesthesia/surgery group, as show in Figures 4B–D. No significant difference was found for p-mTOR, LC3-II and Beclin-1 levels between Con group and Con+Rapa (*p* > 0.05; Figure 4). Notably, mTOR inhibition by rapamycin administration was able to restore p-mTOR increase and LC3-II and Beclin-1 reduction induced by anesthesia/surgery in aged mice.

### Rapamycin Pretreatment Increased SYN and PSD-95 Levels in the Hippocampus

**Figure 5:**
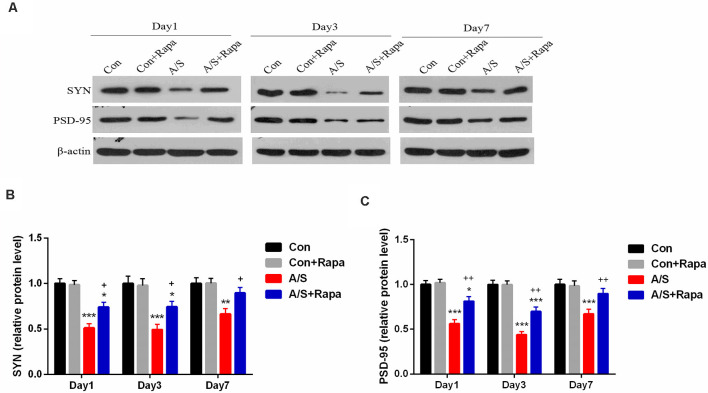
Rapamycin treatment increased SYN and PSD-95 expression in the hippocampus. **(A)** Representative western blot illustrating the effects of rapamycin on the anesthesia/surgery-induced changes in hippocampal SYN and PSD-95 levels on days 1, 3 and 7 after anesthesia/surgery. **(B,C)** Rapamycin reversed anesthesia/surgery-induced decrease in SYN and PSD-95 levels in the hippocampus on days 1, 3 and 7 after anesthesia/surgery. Data are mean ± SEM (*n* = 6 per group). ***p < 0.001, **p < 0.01, *p < 0.05 vs. Con. ^++^*p* < 0.01, ^+^*p* < 0.05 vs. A/S. Con, control group; Con+Rapa, control group with rapamycin pretreatment; A/S, anesthesia/surgery group; A/S+Rapa, anesthesia/surgery group combined with rapamycin pretreatment.

In order to investigate whether the hyperactivation of p-mTOR was involved in the reduction of SYN and PSD-95, the present study examined the effect of rapamycin on the levels of SYN and PSD-95 in the homogenates of the hippocampal tissue using western blot analysis (Figure 5A). It was found that rapamycin significantly increased the production of SYN (*F*_(3,60)_ = 35.35; *p* < 0.001) and PSD-95 (*F*_(3,60)_ = 54.66; *p* < 0.001) on days 1, 3 and 7 after operation by using two-way ANOVA, compared with the anesthesia/surgery group, as shown in Figures 5B,C. There was no difference in the levels of SYN and PSD-95 between Con group and Con+Rapa (*p* > 0.05; Figure 5). Notably, rapamycin preadministration was capable of restoring the SYN and PSD-95 levels.

### Anesthesia/Surgery and Treatment With Rapamycin Were Not Significantly Affected the mRNA Levels of SYN and PSD-95 in the Hippocampus

**Figure 6:**
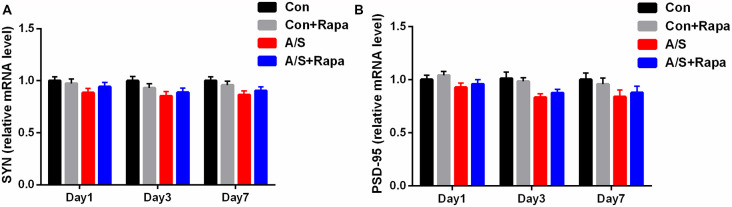
Anesthesia/surgery and treatment with rapamycin were not significantly affected the mRNA levels of SYN and PSD-95. **(A,B)** SYN and PSD-95 mRNA levels in the hippocampus on days 1, 3, and 7 after anesthesia/surgery. Data are mean ± SEM (*n* = 6 per group). Con, control group; Con+Rapa, control group with rapamycin pretreatment; A/S, anesthesia/surgery group; A/S+Rapa, anesthesia/surgery group combined with rapamycin pretreatment.

The mRNA levels of SYN and PSD-95 in the hippocampus were measured by qRT-PCR on days 1, 3 and 7 after anesthesia/surgery. No significant change was shown in mRNA levels of SYN and PSD-95 in the hippocampus at different time points after anesthesia/surgery (*p* > 0.05; Figure 6). These data indicated that treatment with rapamycin did not significantly increase the mRNA levels of SYN and PSD-95 in the hippocampus in aged mice.

### Rapamycin Alleviated the Negative Effects of Anesthesia/Surgery on Reference Memory

**Figure 7:**
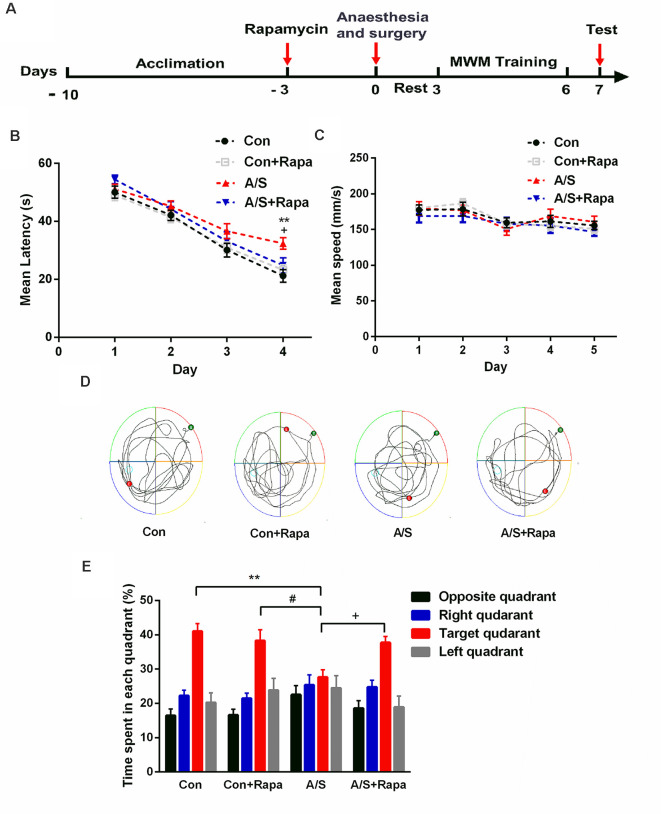
Pretreatment with rapamycin alleviated the effects of anesthesia/surgery on reference memory in aged mice. **(A)** Schematic timeline of the experimental paradigm of surgery procedure and MWM test. After 3 days treatment with rapamycin, exploratory laparotomy was performed, training was conducted for 4 days followed by probe tests on day 7. **(B)** Escape latency to reach the hidden platform during the 4-day training; rapamycin reversed anesthesia/surgery-induced increase in escape latency. **(C)** Average swimming speed; there was no significant difference among the three groups. **(D)** Representative exploratory path of four groups of mice in the probe test. **(E)** The percentage of time spent in the target quadrant during the probe test; rapamycin reversed anesthesia/surgery-induced decrease in time spent in the target quadrant. Data are mean ± SEM (*n* = 12 per group). **p < 0.01 vs. Con. ^#^*p* < 0.05 vs. Con+Rapa. ^+^*p* < 0.05 vs. A/S. Con, control group; Con+Rapa, control group with rapamycin pretreatment; A/S, anesthesia/surgery group; A/S+Rapa, anesthesia/surgery group combined with rapamycin pretreatment.

Previous studies demonstrated that the increased p-mTOR contributed to the cognitive dysfunction caused by anesthesia/surgery. The effect of rapamycin, the mTOR inhibitor, administration on learning and memory function was determined using a MWM assessment, which was also used to measure hippocampal-dependent learning and memory. After 3 days treatment with rapamycin, exploratory laparotomy was performed. After 2 days of rest, we evaluated spatial reference memory with the Morris water maze (Figure 7A). As the training days increased, the mean latency of four groups decreased; and it was significantly higher in the anesthesia/surgery group than in the other three groups on the fourth day of the training period (*F*_(3,176)_ = 5.846, *p* < 0.001; Figure 7B). The average swimming speed during the training and probe tests was not significantly different among the four groups (*p* > 0.05; Figure 7C; exploratory path of four groups of mice in the probe test; Figure 7D). During the probe test, mice given rapamycin exhibited a similar preference for the target quadrant as the control group. Meanwhile, mice in the rapamycin + anesthesia/surgery group could significantly reverse the compromised preference for target quadrant; data compared with two-way ANOVA (*F*_(3,176)_ = 37.04, *p* < 0.001; Figure 7E). These data suggested that rapamycin treatment rescued the impairment of hippocampal-dependent memory induced by anesthesia/surgery.

## Discussion

In this study, anesthesia and surgery induced hyperactivation of mTOR and decreased the levels of autophagy related proteins including Beclin-1 and LC3-II in the hippocampus. The inhibition of autophagy, reduction of neuronal/synaptic plasticity-related proteins such as SYN and PSD-95 were involved in memory and cognitive impairment. Inhibiting the mTOR hyperactivation with rapamycin administration improved anesthesia/surgery-induced memory and cognitive dysfunction by activating autophagy and increasing synaptic plasticity related proteins SYN and PSD-95 expression. Thus, this present study suggests that inhibiting excessive activity of mTOR and activating autophagy may provide a restorative method for the treatment of anesthesia/surgery-induced cognitive impairment.

The Morris water maze is a common test of cognitive function, which can objectively reflect the spatial learning and memory ability of animals. The swimming speeds during the training and probe tests were comparable between the control mice and those from the surgery group, this reduced interference to the test results. In this study, the avoidance latency period was used to judge the spatial learning ability of mice, time spent in the target quadrant was used to judge the spatial memory ability of mice. Our results showed that during the training course, the mice from the anesthesia/surgery group showed longer escape latency compared with the mice in the control group. During the probe test, the preference for the target quadrant was significantly receded in mice that had undergone anesthesia/surgery compared to control mice. These results suggested that anesthesia/surgery impaired reference memory in aged mice. mTOR inhibitor (rapamycin) pretreatment significantly compromised the decreased preference for target quadrant caused by anesthesia/surgery. During the training course, the escape latency of mice in the A/S+Rapa group was significantly higher on day 4 than the A/S group. These data suggested that anesthesia/surgery induced the hyperactivity of mTOR, which was involved in the postoperative cognitive deficits.

The mammalian target of rapamycin (mTOR) is an atypical serine/threonine kinase, and it is critical in cell growth, proliferation, protein synthesis, autophagy (Laplante and Sabatini, 2012; Efeyan et al., 2015). Autophagy is utilized by cells to clear damaged proteins which play an essential role in the neurodegenerative diseases (Scrivo et al., 2018; Djajadikerta et al., 2020). With regard to autophagy, Beclin-1 is considered as a common index for detecting autophagy. Beclin-1 plays a key role in triggering the formation of autophagosome and is essential for the recruitment of other autophagy-related proteins (Sinha et al., 2008). In the AD mice, Beclin-1 expression and the number of autophagosome were significantly decreased, however, increasing Beclin-1 expression promoted the formation of autophagosomes and reduced the aggregation of Aβ (Singh et al., 2017). Microtubule-associated protein 1 light chain 3 (LC3), another specific gene to autophagy in mammalian, and expression of LC3-II is regarded as a marker of the activity of autophagy (Shpilka et al., 2011). Therefore, this study detected the expression of Beclin-1 and LC3-II, which were related to autophagy. Previous studies have suggested that the process of autophagy is negatively regulated by the activation of mTOR (Kim et al., 2011; Alers et al., 2012; Abada and Elazar, 2014). Numerous studies have shown that the role of mTOR signaling in protein homeostasis appears to be particularly important in learning and memory function (Bavley et al., 2018; Gao et al., 2018; Yuan et al., 2019). In the present study, we found that the levels of p-mTOR increased and Beclin-1 and LC3-II decreased in the hippocampus of mice undergone anesthesia/surgery. Furthermore, we showed that activation of autophagy following the administration of rapamycin reversed the cognitive deficits induced by anesthesia/surgery. Jiang et al. (2020) found that the levels of p-mTOR was increased following surgical trauma, an inhibitor of mTOR, rapamycin promoted autophagy activation. These data indicate that the mTOR-inhibited autophagy activation is involved in cognitive impairment induced by anesthesia/surgery.

Numerous studies have suggested that autophagy is involved in synaptic plasticity and neurotransmission (Hernandez et al., 2012; Nibuya et al., 2014; Takahashi et al., 2014). Autophagy-related synaptic dysfunction is associated with neurodegeneration, including Parkinson’s disease and Alzheimer’s (Harris and Rubinsztein, 2011; Nixon, 2013; Vijayan and Verstreken, 2017). Autophagy maintains homeostasis of synaptic proteins (Levine and Kroemer, 2019). Previous studies have shown that the age-related decline in memory is associated with a downregulation of ATG proteins (Shibata et al., 2006; Cuervo, 2008; Rubinsztein et al., 2011). Thus, autophagy is vital to the homeostasis of synaptic proteins and memory consolidation (Shehata et al., 2012; Nixon, 2013). Glatigny et al. (2019) demonstrated that stimulation-induced autophagy was necessary to form new memories in the hippocampus and enhanced dendritic spine density and GluA1 and CAMKII a phosphorylation, markers of synaptic plasticity. Synaptic plasticity is considered important in learning and memory (Howland and Wang, 2008; Tian et al., 2015; Mansvelder et al., 2019). SYN and PSD-95 are involved in neuronal growth, process formation and synaptic plasticity regulation (Sifonios et al., 2009; Li et al., 2016) and play a key regulatory role in normal signal transmission among neurons in the central nervous system (Marco et al., 2017). Liu et al. (2017) demonstrated that cerebrolysin alleviated cognitive deficits induced by increasing the levels of synaptic plasticity-related proteins in the rat hippocampus, such as postsynaptic density protein 95 (PSD-95), protein kinase C subunit gamma (PKCγ). Tian et al. (2016) demonstrated that resveratrol limited diabetes-associated cognitive decline in rats by modulating hippocampal structural synaptic plasticity and enhancing SYN and GAP-43 expression in the hippocampus.

Our results showed that the levels of Beclin-1 and LC3-II, which are related to autophagy, and neuronal/synaptic plasticity-related proteins including SYN and PSD-95 decreased in the hippocampus of mice following anesthesia/surgery. At the same time, the results from the present study demonstrated the effect of rapamycin pretreatment on activation of autophagy, Beclin-1 and LC3-II expression was significantly up-regulated in the hippocampus, meanwhile, the levels of SYN and PSD-95 also increased. Thus, the inhibition of autophagy may probably be responsible for the hippocampal decline of SYN and PSD-95 following anesthesia and surgery.

Our study does have limitations. First, we did not identify specific mechanism of autophagy regulating SYN and PSD-95 in the anesthesia/surgery-induced cognitive decline. Further investigations are necessary to determine the specific molecular mechanisms between synaptic plasticity and autophagy. Second, rapamycin, as an immunosuppressive drug clinically, was observed and tested only for 7 days after anesthesia/surgery. The long-term effects of rapamycin on cognitive impairment after anesthesia/surgery need to be further explored.

In conclusion, our study demonstrated that anesthesia and surgery led tomTOR hyperactivity in the hippocampus, and disturbed neuronal/synaptic plasticity-related proteins homeostasis, possibly by inhibiting autophagy. These sequential pathological events may ultimately cause cognitive impairment. The results of the present study indicate that a novel signaling transduction mechanism is related to cognitive impairment after anesthesia/surgery. Rapamycin may be a potentially therapeutic agent for the treatment of anesthesia/surgery-induced cognitive impairment.

## Data Availability Statement

The original contributions presented in the study are included in the article, further inquiries can be directed to the corresponding author.

## Ethics Statement

The animal study was reviewed and approved by the Animal Care and Use Committee of the Second Affiliated Hospital of Jiaxing University.

## Author Contributions

SG, SZ, HZ and JL designed the experiments. SG, SZ, XT, DP and WY performed the experiments. XT, YN, DP and SK contributed to data analyses. SG drafted this manuscript. HZ and JL reviewed and edited the manuscript. All authors interpreted the data, revised the manuscript, approved the final content, and read and approved the final manuscript. All authors contributed to the article and approved the submitted version.

## Conflict of Interest

The authors declare that the research was conducted in the absence of any commercial or financial relationships that could be construed as a potential conflict of interest.
